# Effects of Leg Stiffness Regulated by Different Landing Styles on Vertical Drop Jump Performance

**DOI:** 10.2478/hukin-2022-0066

**Published:** 2022-09-08

**Authors:** Chieh-Hsin You, Chi-Huang Huang

**Affiliations:** 1 Graduate Institute of Sports Science, National Taiwan Sport University, Taoyuan, Taiwan.; 2 Department of Athletic Training and Health, National Taiwan Sport University, Taoyuan, Taiwan.

**Keywords:** stretch-shortening cycle, stiff, soft, power, reactive strength

## Abstract

The purpose of this study was to investigate the effects of stiffness regulated by landing styles on drop jump performance. Twenty-four male lacrosse athletes performed drop jumps with stiff (ST), self-selected (SS), and soft (SF) landing from a 0.42 m box. Leg stiffness, ground contact time, depth, jump height, maximum ground reaction force (GRF), GRF at the start of the propulsive phase, mean power, peak power, and the reactive strength index (RSI) were calculated. The results showed that jump height and the RSI had strong correlations to power production in all drop jump styles. Power would be a key factor to overall athletic performance. Repeated measures ANOVA showed significant differences (p < 0.05) in all variables among the three styles. Drop jumps with SS landing had comparable jump height to drop jumps with SF landing and power output to drop jumps with ST landing. Drop jumps with ST landing had significantly lower jump height, but higher GRF, power, and the RSI compared to drop jumps with SF landing. In drop jump testing, drop jumps with SS landing should be used if power and jump height were the major concerns; if the RSI was the major concern, drop jumps with ST landing should be used. Training with drop jumps, one of the main objectives should be increasing power output due to its significant correlation to jump height and the RSI in all conditions.

## Introduction

The drop jump is frequently used as a training exercise as well as a test for power and reactive strength in athletic development programs. The ability to express high force and power is essential for athletic performance in many sports. Plyometric exercises that utilize the stretch-shortening cycle (SSC) are commonly used as a method to increase force and/or power output (Slimani et al., 2016). Increased eccentric preloading in the SSC, when appropriate in magnitude, has been shown to improve concentric power output (Bobbert et al., 1987a; Gillen et al., 2019; Voigt et al., 1995; Walsh et al., 2004). Several mechanisms have been proposed to explain the role of eccentric preloading in augmenting jump performances, such as utilization of elastic energy stored in the muscle-tendon complex (Kubo et al., 2000; Voigt et al., 1995), a higher level of active state (Bobbert et al., 1996), pre-programmed activation (Hobara et al., 2007), and stretch-reflex (Komi and Gollhofer, 1997).

As a training exercise, the drop jump is aimed at improving various strength qualities and power output (Markovic and Newton, 2007; Marshall and Moran, 2013; Young et al., 1999). Several performance indices can be extracted from a drop jump test, such as jump height, force, power, the reactive strength index (RSI), etc. (Barr and Nolte, 2011; Bobbert et al., 1987b; Young et al., 1995). Therefore, the drop jump can be used to assess the ability to utilize the SSC in field sports (Barr and Nolte, 2011; Young et al., 2002).

The magnitude of preloading in a drop jump can be affected by the height of the free fall (Bobbert et al., 1987b; Gillen et al., 2019; Voigt et al., 1995; Walsh et al., 2004). In addition to drop height, the landing technique may also have its impact on the magnitude of preloading (Bobbert et al., 1987a; Marshall and Moran, 2013; Walsh et al., 2004). Stiff and soft drop landing have been defined by the angle of maximal knee flexion (Devita and Skelly, 1992). Verbal cue have also been used to alter the landing technique (Myers et al., 2011).

The variance in the technique when performing a drop jump would result in distinct mechanical characteristics. Walsh et al. (2004) reported higher ground reaction forces in stiffer drop jumps and the greatest power output occurred in moderate stiffness. Young et al. (1995) demonstrated that a softer drop jump with longer contact time would result in greater jump height. Guy-Cherry et al. (2018) compared the effects of soft, self-selected, and stiff style drop jumps and showed that the ground reaction forces (GRFs) were significantly different between styles and the soft drop jump had the smallest RSI. Since GRF, power, and the RSI had different responses on the stiffness of drop jumps, it is imperative to understand how the techniques of drop jumps affect various performance variables to choose the appropriate drop jump style in assessment or training for the intended feature. Therefore, a thorough investigation of the effect of drop jump landing styles is needed to understand its effect on athletic performance.

Since the drop jump could be used to assess and develop athletic performance with augmented eccentric preloading, understanding the effects of landing styles on drop jump performance may help practitioners select proper instructions for the intended feature in testing and training. This study measured mechanical variables including leg stiffness, contact time, depth, jump height, GRF_max_, GRF at the start of the propulsive phase (GRF_bottom_), average propulsive power (P_avg_), peak propulsive power (P_peak_), and the RSI in drop jumps with soft (SF), self-selected (SS), and stiff (ST) landing styles. The primary purpose was to examine the correlations between contact time, depth, force, jump height, power and the RSI in each style to determine the major factor of jump performance. The secondary purpose of this study was to investigate the changes of mechanical variables with different drop jump styles. We hypothesized that when landing technique was restricted the ability to exert force and power was correlated to jump performance, while contact time to exert force only had a minor effect. In addition, we hypothesized that drop jump stiffness could be altered by different landing technique and a stiffer jump would have better reactive strength performance, while a softer jump would have better jump height.

## Methods

### Participants

Twenty-four male Taiwanese national team lacrosse players (age: 20.1 *±* 2.7 years; body height: 1.721 *±* 0.049 m; body mass: 73.6 *±* 12.4 kg) were recruited for this study. All participants read and signed an informed consent form approved by the Institutional Review Board of the Fu Jen Catholic University in Taiwan. For participants who were under the age of 18, parental or guardian signed consent was obtained.

### Measures

This research examined drop jumps with three different styles of landing, i.e., SF, SS, and ST. Soft landing was expected to be with the knee most bent and longest ground contact in the three landing styles, while ST landing with the knee least bent and shortest ground contact. The instructions for executing the three landing styles were given using verbal cues. When preforming drop jumps with ST landing participants were told to ”land erect as possible” and ”with little bending at the knee and the hip” and in drop jumps with SF landing they were told to ”land as quietly as possible” and ”use your legs as shock absorbers” as suggested by Myers et al. (2011) with regard to drop landing. Self-selected landing was the landing style that felt most natural and preferred by participants. Two single axis force plates (PS-2141, PASCO Scientific, Roseville, CA) were used to collect GRF data during drop jump landing and the body weight of each participant.

The vertical GRF was collected using PASCO Capstone software (PASCO Scientific) with a sample rate of 1 kHz. The raw data were exported and stored on a personal computer and analyzed with a custom code written in Python (version 3.7). The force signals for each force plate were summed to obtain the overall GRF acting on the body for further analysis. Body weight (BW) was collected as the average vertical forces in the first 0.3 s.

### Design and Procedures

Each participant performed 5 trials of drop jumps with SF, SS and ST landing. The styles executed were arranged in randomized order. After proper warm-up procedures, participants were instructed about the techniques and given time to get familiar with the movements. Before each drop jump was executed, the participant was asked to stand still on the force plates while minimizing body motions for at least 2 s to measure body weight. Then, the participant stepped onto a bench with 0.42 m height and started from standing neutral at the edge of the bench. The participant then executed the drop jump with hands placed on the waist to exclude the effect of the arm swing. Landing was performed with each foot on a force plate followed by a jump as high as possible immediately after landing.

The ground reaction force threshold identifying landing and takeoff was set to be 50 times the standard deviation of force values from 0.05 to 0.25 s after the participant left force plates. Contact time was the duration between the first landing and takeoff. The upward direction was designated as positive in the calculation. The vertical center of mass velocity immediately before ground contact (*v*_0_) was calculated with the following formula:


$$v_{0}=-\sqrt{2gH}$$

where *H* = drop height. Velocity (*v*) at each sample point was calculated with the net impulse and BW:

v = v_0_ + Jnet/(BW/g),

where *J*_net_ = net impulse. The net impulse was the GRF impulse subtracted by the BW impulse. The ground contact was divided into braking and propulsive phases with the onset of the propulsive phase being the first sample point where *v >* 0. With the vertical velocity at each sample point we obtained vertical displacement (*z*) using the following formula:


$$z_{i}=\sum_{s=0}^{i}v_{s}\Delta t$$

where Δ*t* is the time interval between sample points. Work (*W*) and power (*P*) of

GRF can be calculated with the following formula: $W_{i}=\sum_{s=0}^{i}\frac{\text{GR}{\text{F}}_{s}+\text{GR}{\text{F}}_{s-1}}{2}\left[Z_{s}-Z_{s-1}\right],$when s > 0, and


$$P_{i}=\frac{W_{i}-W_{i-1}}{\Delta t}$$

Jump height was the greatest vertical center of mass displacement from the ground. The reactive strength index was jump height divided by the ground contact time in first landing. Drop jump depth was designated as the smallest negative vertical center of mass displacement after first landing. Leg stiffness was determined by a spring-mass model (Ward et al., 2019) and was calculated using the following formula:


$$\text{Stiffness}=\frac{\text{GR}{\text{F}}_{\text{max}}}{\text{Depth}}$$

Force, impulse, power, and stiffness were normalized to body mass.

### Statistical Analysis

Time to maximum braking GRF (GRF_max,braking_) and maximum propulsive GRF (GRF_max,propulsive_) were recorded and correlated to time to GRF_max_ to determine which phase had greatest loading in drop jump landing. Jump height, depth, contact time, GRF_max_, GRF_bottom_, P_avg_, P_peak_, and the RSI were recorded for further analysis. The Pearson correlation was calculated within drop jump styles to determine the relationship between each of the variables. The mechanical variables were averaged with five trials and compared between landing styles using repeated measures one-way analysis of variance (RepANOVA). Normality of distribution of each averaged variable was tested using the Shapiro-Wilk test. Post-hoc analysis was performed using the *t*-test with Bonferroni corrections if the result of RepANOVA was statistically significant. The Alpha level was set at .05 for statistical significance. Effect size (Cohen’s d) of the drop jump styles was reported and the magnitude was classified as: trivial (<0.20), small (0.20–0.50), moderate (0.51–0.80) or large (>0.80). The power of RepANOVA was .74 for a moderate effect size .25 from an a priori statistical power analysis using G*Power (version 3.1, Heinrich-Heine-Universität Düsseldorf, Germany). The remaining statistical analyses were performed using Python (version 3.7) with *statsmodels* and *scipy* modules.

## Results

**Figure 1:**
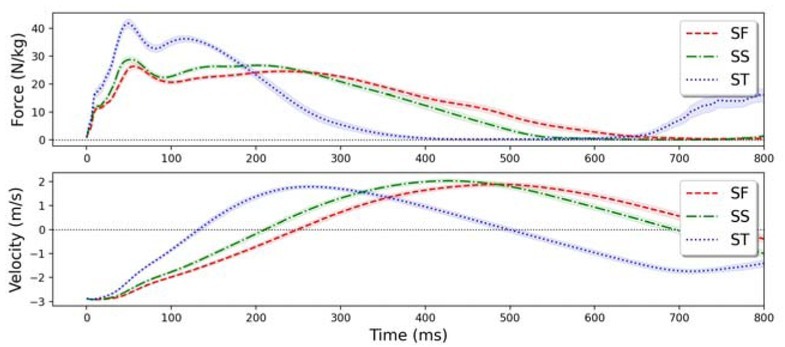
Ensemble data of each drop jump style. Shaded areas show 95% confidence intervals.

Figure 1 shows the means and confidence intervals of force-time and velocity-time curves. Time to GRF_max,braking_ was strongly correlated to time to GRF_max_ (*r* = .998, *p* < .01) and had a slope close to 1, while time to GRF_max,propulsive_ was moderately correlated to time to GRF_max_ (*r* = .246, *p* < .01) (Table 1).

**Table 1:** Correlations of each time variables for GRF

	Time to GRF_max_	
	Slope	R^2^
Time to GRF_max,braking_	0.999	0.996^*^
Time to GRF_max,propulsive_	0.197	0.061^*^

Note: ^*^p < .001.

Table 2 summarizes the correlations between variables in different conditions. Peak and average propulsive power had strong and significant correlations to jump height and the RSI (*r* = .755-.986, *p* < .001). Jump height was moderately correlated to contact time in drop jumps with SF and ST landing (*r* = .268-.345, *p* < .01), but not in drop jumps with SS landing (*p* = .627). Among the three styles, GRF_bottom_ was correlated to jump height only in drop jumps with SS landing (*r* = .336, *p* < .01). There were significant correlations between power (P_avg_ & P_peak_) and GRF_bottom_ (*r* = .285-.671, *p* < .01). Correlations between contact time and the RSI were moderate to strong in (*|r|* = .366-.624, *p* < .001). The RSI had strong correlations to jump height and GRF_bottom_ (*r* = .512-.735, *p* < .001).

**Table 2:** Correlations (r) between kinetic and kinematic variables

		Soft	Self-Selected	Stiff
Jump height	Contact time	0.268^*^	0.045	0.345^*,†^
Jump height	Depth	0.280^*^	0.066	0.226
Jump height	GRF_max_	-0.151	-0.026	-0.094
Jump height	GRF_bottom_	0.228	0.336^*,†^	-0.156
Jump height	P_avg_	0.755^*,†^	0.814^*,†^	0.783^*,†^
Jump height	P_peak_	0.845^*,†^	0.795^*,†^	0.790^*,†^
P_avg_	GRF_max_	0.058	0.275^*^	0.179
P_avg_	GRF_bottom_	0.637^*,†^	0.671^*,†^	0.459^*,†^
P_peak_	GRF_max_	0.044	0.247^*^	0.141
P_peak_	GRF_bottom_	0.285^*^	0.474^*,†^	0.431^*,†^
RSI	Jump height	0.683^*,†^	0.735^*,†^	0.730^*,†^
RSI	Contact time	-0.502^*,†^	-0.624^*,†^	-0.366^*,†^
RSI	P_avg_	0.971^*,†^	0.975^*,†^	0.986^*,†^
RSI	P_peak_	0.885^*,†^	0.943^*,†^	0.964^*,†^
RSI	GRF_max_	0.142	0.386^*,†^	0.278^*^
RSI	GRF_bottom_	0.592^*,†^	0.639^*,†^	0.512^*,†^

Note: ^*^p < .01 ^†^p < .001

**Figure 2:**
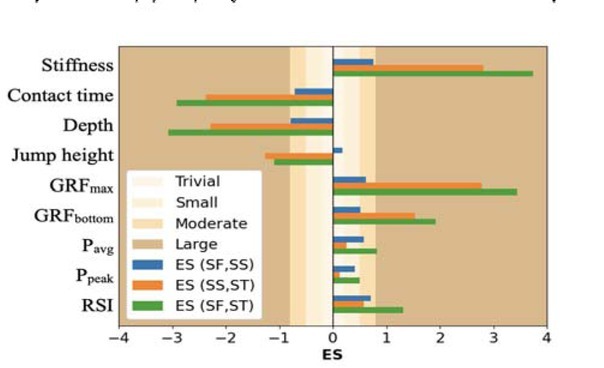
Cohen’s d effect size (ES) of drop jump styles on mechanical variables in Table 3.

The drop jump styles had significant effects (*p* < .05) on all mechanical variables presented in Table 3. Cohen’s *d* effect size of different drop jump styles on mechanical variables is shown in Figure 2. All variables shown in Table 3 were normally distributed which was checked using the Shapiro-Wilk test (*p* > .05). P_avg_ and P_peak_ increased from drop jumps with SF landing to drop jumps with SS landing with no further differences in drop jumps with ST landing. For jump height, there were no differences between drop jumps with SF and SS landing and a decrease in drop jumps with ST landing. Both GRF_max_ and GRF_bottom_ increased systematically from drop jumps with SF landing to drop jumps with ST landing; however, the increase was not significant in GRF_bottom_ from drop jumps with SF landing to drop jumps with SS landing. The RSI increased systematically as the drop jump style changed from SF to ST landing. Leg stiffness increased when drop jumps changed from SF to SS and then to ST landing styles. Ground contact time and depth decreased with stiffer drop jumps.

**Table 3:** Mechanical variables of each landing style of drop jumps

	Soft	Self-Selected	Stiff
Stiffness, N/m^*^	82.36 (25.31)	107.71^†^ (39.85)	243.28^†, ‡^ (55.33)
Contact time, s^*^	0.52 (0.1)	0.45^†^ (0.08)	0.29^†,‡^ (0.05)
Depth, m^*^	0.43 (0.09)	0.37^†^ (0.08)	0.23^†,‡^ (0.04)
Jump height, m^*^	0.3 (0.06)	0.31 (0.07)	0.23^†,‡^ (0.06)
GRF_max_, N*·*kg*^-^*^1*^	33.13 (4.81)	36.26^†^ (5.26)	52.55^†,‡^ (6.38)
GRF_bottom_, N*·*kg*^-^*^1*^	26.22 (3.16)	28.11 (4.12)	37.61^†,‡^ (7.74)
P_avg_, N*·*m*·*s*^-^*^1^*·*kg*^-^*^1*^	26.58 (3.87)	29.05^†^ (4.58)	30.28^†^ (5.04)
P_peak_, N*·*m*·*s*^-^*^1^*·*kg*^-^*^1*^	47.59 (5.82)	50.17^†^ (6.55)	51.07^†^ (7.97)
RSI, m*·*s*^-^*^1*^	0.59 (0.14)	0.71^†^ (0.19)	0.83^†,‡^ (0.2)

Note: ^*^Significantly different by RepANOVAs. ^†^Significantly different to SF after post-hoc analysis. ^‡^Significantly different to SS after post-hoc analysis.

## Discussion

To our knowledge, this is the first study that investigates the interaction of mechanical variables in different drop jump styles and the effects of the landing style on drop jump performance. Power output depicts the ability to express force during the propulsive phase and thus, had major effects on jump height and the RSI with confined technique rather than ground contact time and depth. Therefore, drop jump height may be used to assess athletes’ power performance when proper instruction is given since it is the key factor of jump height. The correlation between GRF_bottom_ and jump height only existed in drop jumps with SS landing; it may imply that with unfamiliar landing technique the ability to utilize preloading at the very bottom for the following force production was impaired. As it was as expected, the RSI had significant correlations to jump height and ground contact time since these two variables make up the definition of the RSI. Moreover, the RSI had lowest correlation to contact time in drop jumps with ST landing and strong correlation to jump height. This result may suggest that the stiff landing drop jump reflected athletes’ ability of using the SSC to convert eccentric preloading to concentric output most properly among these three styles.

In drop jumps, GRF_max_ generally occurred in the eccentric phase (Makaruk and Sacewicz, 2011; McBride et al., 2008), which can be inferred with the coincidence between the time to GRF_max,braking_ and GRF_max_ and the force-time curve. Although GRF_max,propulsive_ also showed a significant correlation with GRF_max_, yet the correlation was only moderate which indicated that propulsive force was dependent on GRF_max_, but not the maximum force exertion. Therefore, GRF_max_ was not directly related to propulsive power output, which may explain why GRF_max_ did not show a significant correlation to power indices as GRF_bottom_ did. The correlations of GRF_bottom_ to P_avg_ and P_peak_ may be explained by the fact that greater GRF at the start of the propulsive phase could lead to a higher overall force level and thus, higher power output. This result highlighted the importance of the strength level in optimizing power which was also stated in the work by Haff and Nimphius (2012).

The tendencies of increasing leg stiffness along with decreasing ground contact time and depth from SF to ST landing indicated the efficacy of altering landing techniques with verbal cues in this study. The main finding of RepANOVA was drop jumps with SF and SS landing had greater jump height, while drop jumps with SS and ST landing had higher P_peak_ and P_avg_. Although drop jumps with ST landing had greater GRF_max_ and GRF_bottom_, contact time was shorter and thus, jump height determined by the ground reaction impulse had a significant drop in drop jumps with ST landing. The increase in GRF with stiffer drop jumps resulted in higher P_avg_ and P_peak_ in drop jumps with SS landing. However, no further increases in P_avg_ and P_peak_ were observed in drop jumps with ST landing. This might be due to the slower takeoff velocity in drop jumps with ST landing that prevented greater power output even with higher GRF. Thus, the resultant power was similar to that in drop jumps with SS landing. This result is similar to previous findings which showed that when drop jumps went stiffer, there would be a point beyond which there would be no concurrent increase in jump height and power output with force (Walsh et al., 2004). Although there were different trends between jump height and power, drop jumps with SS landing seemed to be the best strategy to optimize jump height and power at the same time. As previously reported, the drop jump technique that resulted in shorter contact time was accompanied with a greater RSI, but lower jump height (Struzik et al., 2016; Young et al., 1995). In this study, RSI values were significantly different in all three groups, which might indicate that by altering the drop jump technique, changes in contact time had greater effect on the RSI than changes in jump height.

Comparing results of current research to previous studies, the RSI was comparable to that reported by Guy-Cherry et al. (2018) (SF: 0.7 vs. 0.6; SS: 0.7 vs. 0.9; ST: 0.8 vs. 0.9) with similar experimental setup including drop height and technique used. Other studies (Barr and Nolte, 2011; Young et al., 2002) showed dissimilar results. Ground contact times of all three groups in this study were longer than those in the research by Walsh et al. (2004). The reasons may be a different method used, e.g. force plates vs. a contact mat, different drop height, technique instructions or differences in the strength level of participants since there were younger athletes with less experience in strength and jump training in this study.

In a drop jump test, the technique used should manifest the targeted feature. Self-selected drop jumps seem to be appropriate for testing power production since power was greatest and the influence on jump height was highest in drop jumps with SS landing. To assess reactive strength, drop jumps with ST landing are the suggested method because they exhibited best RSI values and thus, presented athletes’ ability to utilize a fast SSC. Neither GRF nor power output in drop jumps with SF landing was higher than in the other two styles to elicit best performance in drop jump testing.

In training, drop jumps with SF landing could be used for novice athletes to train under lower stress, but still more strenuous than countermovement jumps due to the larger joint moment (Bobbert et al., 1987a). However, as the athlete becomes more experienced, progressing to drop jumps with SS and ST landing is required to assure enough training stimuli. Using drop jumps with SF/SS landing for jump height and drop jumps with ST landing for reactive strength in training is recommended because these styles demonstrated best performance for the task-specific attributes. Marshall and Moran (2013) reported that after an eight week training intervention with softer drop jumps, countermovement jump height was increased more than that with stiffer drop jumps. Young et al. (1999) showed that training with stiff drop jumps was effective for the RSI. Although these two studies verified the idea of training with task-specific techniques, the longest training period was 8 weeks. It should also be noted that drop jumps with ST landing showed the greatest force and power which imposed greater stress on the body. Therefore, incorporating drop jumps with ST landing in training might be required for stronger athletes to adapt to greater force and power, which correlates to jump height and the RSI in all conditions.

In this study, regression analysis showed that the ability to express power under different preloading schemes was the major factor of jump height and the RSI. Therefore, improving power output may have benefits for the overall athletic performance. Stiffness regulated by the landing technique had impacts on various performance variables. Stiffer drop jumps associated with greater preloading had higher GRF, power, and the RSI. On the other hand, softer drop jumps with lower preloading resulted in greater jump height. With the differences among drop jump styles presented in this study, in training and testing the technique of executing drop jumps should be determined based on the one that yields best targeted performance.
